# Previously unknown quasicrystal periodic approximant found in space

**DOI:** 10.1038/s41598-018-34375-x

**Published:** 2018-11-02

**Authors:** Luca Bindi, Joyce Pham, Paul J. Steinhardt

**Affiliations:** 1 Dipartimento di Scienze della Terra, Università di Firenze, Via La Pira 4, I-50121 Florence, Italy; 2 Max-Planck-Institut für Chemische Physik fester Stoffe, Nöthnitzer Straße 40, 01187 Dresden, Germany; 3 Department of Physics, Princeton University, Jadwin Hall, Princeton, NJ 08544 USA; 4 Princeton Center for Theoretical Science, Princeton University, Princeton, NJ 08544 USA

## Abstract

We report the discovery of Al_34_Ni_9_Fe_2_, the first natural known periodic crystalline approximant to decagonite (Al_71_Ni_24_Fe_5_), a natural quasicrystal composed of a periodic stack of planes with quasiperiodic atomic order and ten-fold symmetry. The new mineral has been approved by the International Mineralogical Association (IMA 2018-038) and officially named proxidecagonite, which derives from its identity to periodic approximant of decagonite. Both decagonite and proxidecagonite were found in fragments from the Khatyrka meteorite. Proxidecagonite is the first natural quasicrystal approximant to be found in the Al-Ni-Fe system. Within this system, the decagonal quasicrystal phase has been reported to transform at ~940 °C to Al_13_(Fe,Ni)_4_, Al_3_(Fe,Ni)_2_ and the liquid phase, and between 800 and 850 °C to Al_13_(Fe,Ni)_4_, Al_3_(Fe,Ni) and Al_3_(Fe,Ni)_2_. The fact that proxidecagonite has not been observed in the laboratory before and formed in a meteorite exposed to high pressures and temperatures during impact-induced shocks suggests that it might be a thermodynamically stable compound at high pressure. The most prominent structural motifs are pseudo-pentagonal symmetry subunits, such as pentagonal bipyramids, that share edges and corners with trigonal bipyramids and which maximize shortest Ni–Al over Ni–Ni contacts.

## Introduction

The first decagonal quasicrystalline (QC) phase found in nature, decagonite Al_71_Ni_24_Fe_5_^1,2^, was discovered in the Khatyrka meteorite, a CV3 carbonaceous chondrite^3–8^. Its structure consists of a periodic stack of planes that each have a crystallographically forbidden ten-fold symmetric, quasiperiodic arrangement of atoms. The finding followed the discovery of icosahedrite Al_63_Cu_24_Fe_13_^9,10^, the first quasicrystal discovered in nature, which displays three-dimensional icosahedral symmetry and is quasiperiodic in all directions. The chemical compositions of both decagonite and icosahedrite match those of the QC phases previously synthesized in a laboratory setting at standard pressure^11,12^.

In the search through Khatyrka meteoritic fragments recovered from a 2011 expedition to the Koryak Mountains in far eastern Russia^3–6^, various other novel intermetallic phases have been uncovered^13–16^, including a second Al-Cu-Fe icosahedral QC phase that differs from icosahedrite and that is the first QC to be discovered in nature before being synthesized in the laboratory^17^. However to date, no natural periodic approximants to quasicrystals have been reported.

Periodic approximant is an accepted technical term that refers to a crystalline solid with similar chemical composition to a QC, but whose atomic arrangement is slightly distorted so that the symmetry conforms to the conventional laws of three-dimensional crystallography. Crystalline approximants can be considered the missing link between QCs and crystals and are very useful because they provide a well-defined starting point for models of the local atomic structure of the corresponding quasicrystals. The discovery of approximants forming at high pressure may indicate the existence and stability of yet more types of QCs at non-standard conditions.

Herein we report the discovery of the first natural periodic approximant to the decagonal QC, Al_71_Ni_24_Fe_5_. The approximant, with chemical formula Al_34_Ni_9_Fe_2_, does not correspond to any previously recognized synthetic^18^ or natural phase. The mineral was named proxidecagonite, derived from “periodic approximant of decagonite” (from the truncated Latin word *proxǐmus* followed by the name of the quasicrystalline mineral decagonite). The new mineral and its name have been approved by the Commission on New Minerals, Nomenclature and Classification of the International Mineralogical Association (IMA 2018-038). The holotype material is deposited in the collections of the Museo di Storia Naturale, Università degli Studi di Firenze, Via La Pira 4, I-50121, Firenze, Italy, catalogue number 3291/I.

Proxidecagonite was found in one of the meteoritic fragments of the same Khatyrka meteorite labeled Grain 126. All recovered fragments of Khatyrka including Grain 126 have been shown to include non-metallic minerals with CV3-like oxygen isotopic compositions^4,7,13,14^ confirming their common meteoritic origin. The Khatyrka meteorite formed 4.5 billion years ago during the earliest stages of the solar system and contains evidence of a heterogeneous distribution of pressures and temperatures during impact shock, in which some portions of the meteorite reached at least 5–10 GPa and 1200–1500 °C. Based on noble gas studies, the most recent strong shock experienced by Khatyrka took place in space a few 100 Ma^8^. Lin *et al*.^7^ have also shown that some of the metallic Al-alloys in Khatyrka pre-dated the shock.

## Results

### Description of the sample

**Figure 1:**
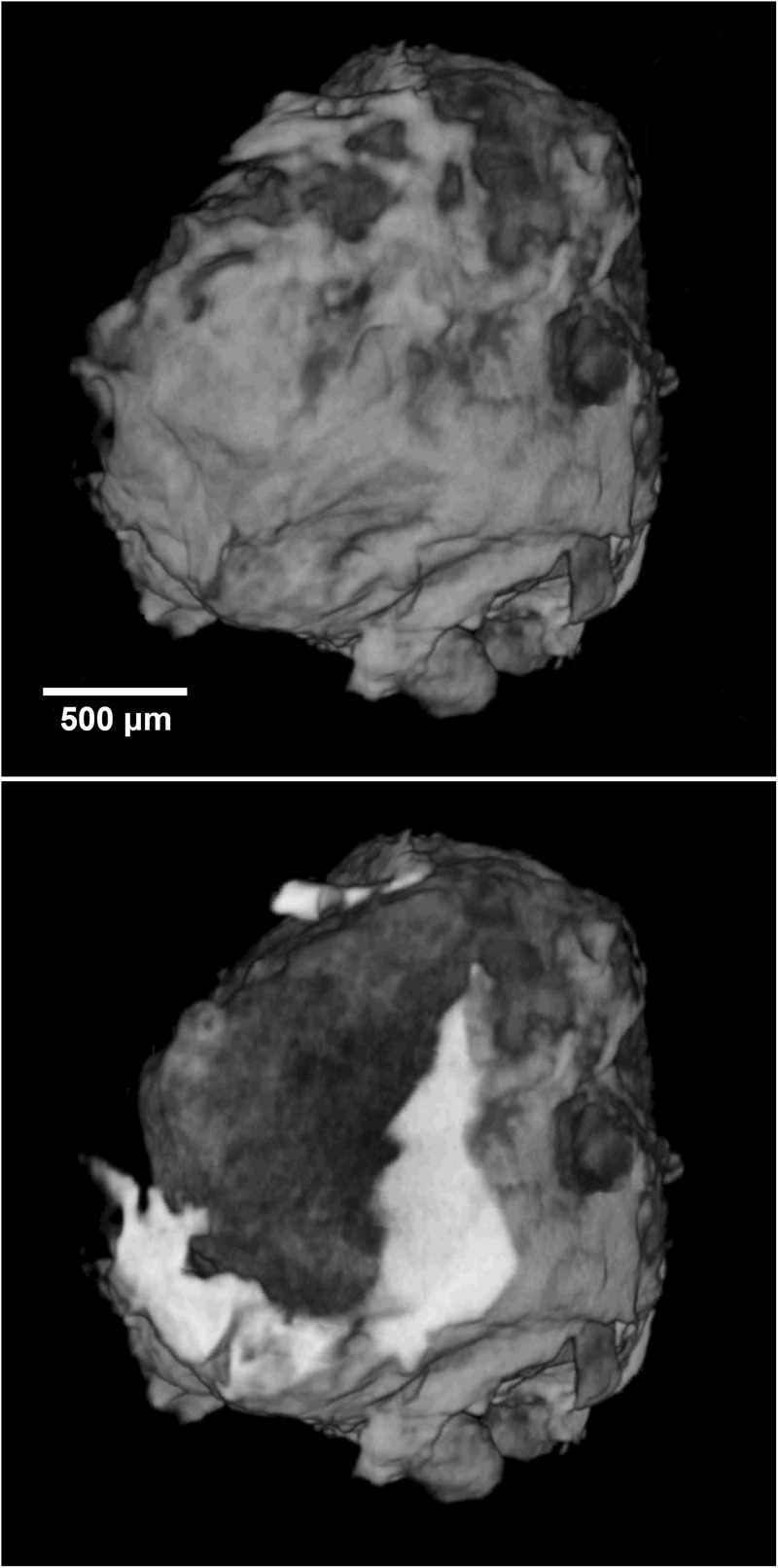
Micro-computed tomographic images (at different orientations) of the entire grain (labeled number 126). Light grey and dark grey regions refer to metallic (AlCu, AlCuFe, AlFe, and AlNiFe minerals) and silicatic portions.

Grain 126 is dark grey in incident light with diverse silicate and metallic fragments visible. No fusion crust is preserved on the sample. X-ray computed tomography studies showed the presence of a large khatyrkite grain (bright areas in the upper panel of Fig. 1) clearly attached to the meteorite fragment (dark areas in the upper panel of Fig. 1), as typically observed for other fragments of the Khatyrka meteorite^4,7,13,14^. Detailed examination by scanning electron microscopy, single-crystal X-ray diffraction, micro-computed tomography and transmission electron microscopy of fragments from Grain 126 associated to proxidecagonite revealed the presence of trevorite, diopside, forsterite, ahrensite, clinoenstatite, nepheline, coesite, stishovite, pentlandite, Cu-bearing troilite, icosahedrite, khatyrkite, taenite, Al-bearing taenite, steinhardtite, decagonite, hollisterite, stolperite and kryachkoite^4,5,7,13,15–17^. The recovery of different Al-Ni-Fe crystalline (steinhardtite) and QC (decagonite) intermetallic phases, motivated a careful search for other metallic fragments, which led to the discovery of a particle with composition close to that of the known Al-Ni-Fe decagonal QC but with different diffraction characteristics.

### Description of the selected Al-Ni-Fe approximant

Proxidecagonite exhibits an anhedral morphology and does not show inclusions of or intergrowths with other minerals. The maximum grain size found to date is ~20 μm. The color is grey to black, the streak is black and the lustre is metallic. In plane-polarized incident light, proxidecagonite is weakly to moderately bireflectant and not pleochroic. Internal reflections are absent. Between crossed polars, the mineral is anisotropic, without characteristic rotation tints. The reflectance was measured in air and the reflectance data (*R*_min_ − *R*_max_ and λ/nm) are 28.8–31.2 (471.1), 29.4–32.0 (548.3), 30.6–32.8 (586.6), and 31.5–33.7 (652.3). The most promising proxidecagonite fragment was hand-picked from a TEM grid and studied by means of electron microprobe and X-ray diffraction techniques. The chemical data (Table 1) provided the empirical formula (based on 45 atoms *pfu*) of Al_33.99_Ni_9.00_Fe_1.98_Si_0.02_Co_0.01_, ideally Al_34_Ni_9_Fe_2_. The fragment was then studied by single-crystal X-ray diffraction. The investigated Al_34_Ni_9_Fe_2_ fragment exhibits the *o*’-Al_13_Co_4_ structure^19^ (orthorhombic, space group *Pnma*) with parameters *a* = 29.013(3), *b* = 8.156(1), *c* = 12.401(2) Å, *V* = 2934.4(7) Å^3^ and *Z* = 4.

**Table 1:** Electron microprobe analyses (wt% of elements, ranges, and standard deviations) of proxidecagonite.

Constituent	wt%	Range	SD	Probe Standard
Al	58.75	57.40–59.21	0.32	Al metal
Ni	33.85	32.92–34.15	0.19	synthetic Ni_3_P
Fe	7.09	6.88–7.35	0.10	synthetic FeS
Mg	0.00	0.00–0.02	0.01	Mg metal
Si	0.03	0.01–0.04	0.01	Si metal
Cr	0.01	0.00–0.03	0.02	Cr metal
P	0.00	0.00–0.01	0.01	synthetic Ni_3_P
Co	0.01	0.00–0.03	0.01	Co metal
Cu	0.01	0.00–0.02	0.01	Cu metal
Ca	0.00	0.00–0.01	0.01	synthetic CaCl_2_
Zn	0.01	0.00–0.02	0.01	synthetic ZnS
S	0.00	0.00–0.01	0.01	synthetic ZnS
Cl	0.00	0.00–0.01	0.01	synthetic CaCl_2_
Total	99.76	98.69–100.42		

### Atomic structure

**Figure 2:**
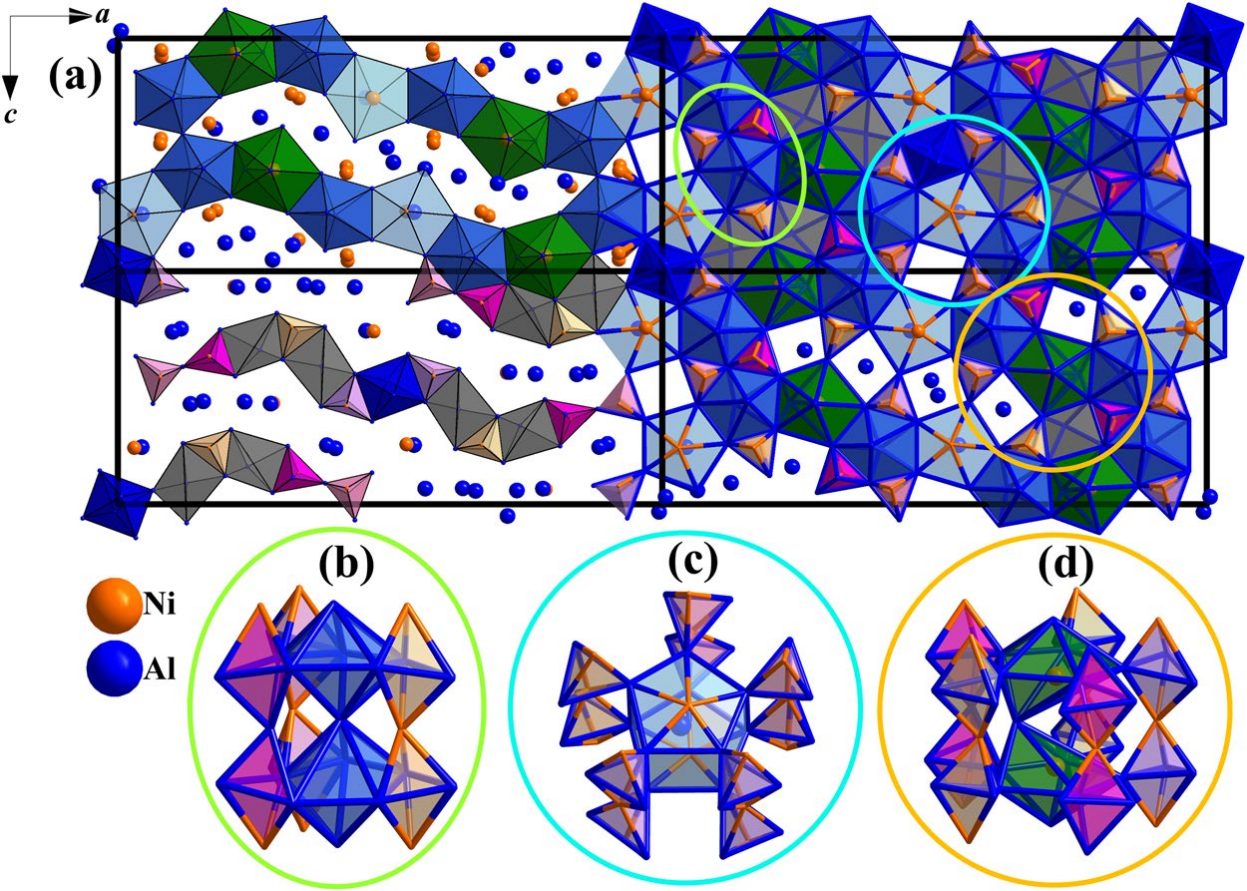
The structure of proxidecagonite refined as “Ni_44_Al_136_” with pseudo-pentagonal symmetry building blocks (**b**–**d**). (**a**) Down the *b*-axis across 4 unit cells: the upper left depicts pseudo-pentagonal symmetry subunits (i.e., distorted pentagonal bipyramids and elongated pentagonal bipyramids) arranged in waves with voids filled by trigonal bipyramids, octahedra, and square pyramids, which are drawn in the lower left unit cell in also a wave-like fashion. The lower right shows trigonal bipyramids and pentagonal subunits sharing edges or corners that are isolated in the inset (**b**–**d**); the upper right shows all polyhedra tiling the unit cell.

The structure of proxidecagonite, in which all Fe and Ni sites were crystallographically refined as Ni so that the composition is “Ni_44_Al_136_”, generally maximizes shortest Ni–Al over Ni–Ni contacts. The atoms are not close-packing, although vacancies have been examined as a contributing stabilization factor in a similar structure in Al_13_Co_4_^20^. Rather than close-packing of the atoms as typically observed in intermetallic compounds, the structure can be described from a close-packing of corner-sharing, empty (non-centered) polyhedra as follows: Al_6_ octahedra, Ni_2_Al_3_ and NiAl_4_ trigonal bipyramids, Al_7_ distorted pentagonal bipyramids, and Al_5_ square pyramids. Figure 2 shows the breakdown of the structure across 4 unit cells with the pseudo-pentagonal symmetry subunits shown in the inset. Moreover, each set of Al-centered Ni_2_Al_10_ elongated pentagonal bipyramids and Ni-centered Al_7_ distorted pentagonal bipyramids share corners with trigonal bipyramids in the pentagonal planes (Fig. 2c,d). On the other hand, the non-centered distorted pentagonal bipyramids share edges with three trigonal bipyramids (Fig. 2b). All polyhedra with pseudo-pentagonal symmetry tile the unit cell in a wave-like manner in the *ac*-plane with voids between them filled by the other polyhedra, which are also arranged in a wave-like fashion, so that Ni atoms intervene for maximal Ni–Al polar-covalent interactions within the structure. A previous description of this structural arrangement in *o*’-Al_13_Co_4_ highlights the pentagonal bipyramids formed by either Al–Al or Co–Co contacts alone^19^, which in “Ni_44_Al_136_” is not sufficient to describe the preference for shortest Ni–Al over Ni–Ni contacts.

### Electronic structure

**Figure 3:**
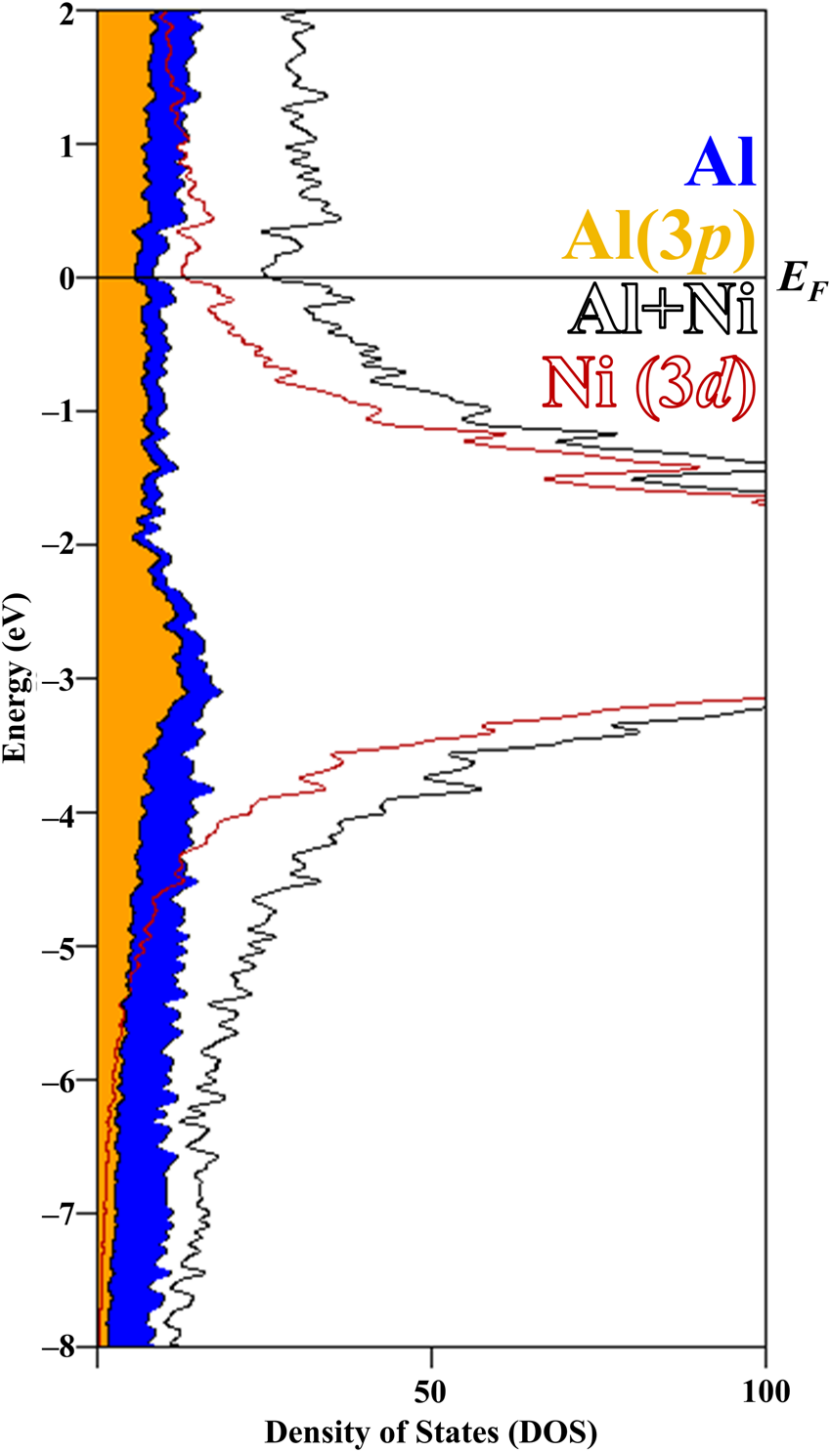
The density of states (DOS) for “Ni_44_Al_136_” with atomic orbital contributions showing Ni 3*d* orbitals interacting most strongly with Al 3*p* orbitals that may give rise to the structural preference to maximize Ni–Al over Ni–Ni shortest contacts. The Fermi energy, *E*_*F*_, sits at a pseudogap, signifying the electronic stability of the crystallographic structure. For the ideal composition Fe_8_Ni_36_Al_136_ with only 16 electrons fewer, *E*_*F*_ sits at the tapering region between the pseudogap and peak.

The density of states (DOS) curves provide a means to rationalize the atomic structure from electronic distributions and atomic orbital interactions. In the DOS of “Ni_44_Al_136_” (Fig. 3), the Fermi energy, *E*_*F*_, sits in a shallow pseudogap, signifying the electronic stability of the crystallographic structure. As part of the rigid-band approximation, which considers the total number of electrons within the system by integrating the DOS and moving *E*_*F*_ along the DOS according to the number of electrons, *E*_*F*_ of the experimentally ideal composition “Fe_8_Ni_36_Al_136_”, with 16 electrons fewer than Ni_44_Al_136_, also sits at the edge of the peak as it tapers to the pseudogap. The Ni 3*d* orbitals dominate below *E*_*F*_ with a width of ~3.5 eV and most strongly interacts with the Al 3*p* orbitals. Near and above *E*_*F*_, the relative contribution of Ni 3*d* orbitals decreases whereas that of Al increases, thus depicting the polar-covalent interactions between the filled Ni(3*d*) and partially filled Al(3*p*) orbitals that give rise to a preference to structurally maximize Ni–Al over Ni–Ni shortest contacts.

## Discussion

The new approximant Al_34_Ni_9_Fe_2_ described here has not been observed as a product of laboratory synthetic experiments, and it represents the second example of a composition discovered in nature prior to discovery in the laboratory. It was found^18^ that at ~940 °C the decagonal QC decagonite (Al_71_Ni_24_Fe_5_) transforms to Al_13_(Fe,Ni)_4_, Al_3_(Fe,Ni)_2_ and the liquid phase, and between 800 and 850 °C to Al_13_(Fe,Ni)_4_, Al_3_(Fe,Ni) and Al_3_(Fe,Ni)_2_. Although Al_13_(Fe,Ni)_4_, the composition with the Al/(Ni + Fe) ratio closest to proxidecagonite (3.25 and 3.09, respectively), shows chemical similarities with proxidecagonite, it crystallizes in the monoclinic Al_13_Fe_4_ structure corresponding to the recently described hollisterite (reported by Ma *et al*.^16^ with the ideal chemical formula Al_3_Fe). Also, Ni dominates significantly over Fe in proxidecagonite, which represents another difference with respect to the other two known synthetic periodic approximants in the Al-Ni-Fe system, i.e. Al_76_Ni_9_Fe_15_ (monoclinic, space group *C*2/*m*^21^) and Al_72_Ni_9_Fe_19_ (hexagonal, *P*6_3_/*mmc*^22^).

Previous work on high pressure phases in the Al-Ni-Fe system were confined to static pressure and did not produce proxidecagonite^23^. Likely to be more relevant are dynamic shock-induced high pressure experiments as it was recently demonstrated that shock conditions may generate previously unknown crystal and QC phases in both the Al-Cu-Fe^24,25^ and Al-Ni-Fe^26^ systems. Based on the examples found thus far, it appears that new unpredicted Ni-rich phases become stabilized over Fe-rich phases at high pressures. By combining studies of natural periodic and aperiodic phases found in Khatyrka with high-pressure diamond anvil experiments^27–,29^ and laboratory shock experiments^24–,26^, we plan to elucidate the kinetic and thermodynamic stability of QCs and discover other novel phases stabilized at high pressure. Aside from their intrinsic interest, the new phases may provide insights on previously unobserved early solar-system processes.

## Methods

### Studied material

Proxidecagonite was investigated by means of SEM (scanning electron microscopy), EMP-WDS (electron microprobe, wavelength dispersive spectrometry), single-crystal and powder X-ray diffraction techniques.

### Scanning electron microscopy

The instrument used was a Zeiss - EVO MA15 Scanning Electron Microscope coupled with an Oxford INCA250 energy-dispersive spectrometer, operating at 25 kV accelerating potential, 500 pA probe current, 2,500 cps as average count rate on the whole spectrum, and a counting time of 500 s. Samples were sputter-coated with 30-nm-thick carbon film.

### Electron microprobe

Quantitative analyses were carried out using a JEOL JXA 8600 microprobe (WDS mode, 15 kV, 10 nA, 1 μm beam size, counting times 20 s for peak and 10 s for background). High spatial resolution was achieved using conditions of 13 kV, 7 nA. For the WDS analyses the *K*α lines for all the elements were used. Variable counting times were used: 30 s for Al, Ni and Fe, and 60 s for the minor elements Mg, Si, Cr, P, Co, Cu, Cl, Ca, Zn, and S. Replicate analyses of synthetic Al_53_Ni_42_Fe_5_ were used to check accuracy and precision. The crystal fragments were found to be homogeneous within analytical error. The standards used were: Al metal, synthetic Ni_3_P (Ni, P), synthetic FeS (Fe), Mg metal, Si metal, Cr metal, Co metal, Cu metal, synthetic CaCl_2_ (Ca, Cl) and synthetic ZnS (Zn, S). Magnesium, Si, Cr, P, Co, Cu, Cl, Ca, Zn, and S were found to be equal to or below the limit of detection (0.01 wt%). Four point analyses on different spots were performed on the three samples. Table 1 reports the chemical data (in wt% of elements) for proxidecagonite.

### Single-crystal X-ray diffraction and structure refinement

Single-crystal X-ray studies were carried out using an Oxford Diffraction Xcalibur 3 diffractometer equipped with an Oxford Diffraction CCD detector, with graphite-monochromatized Mo*K*α radiation (λ = 0.71073 Å), working conditions 50 kV × 50 nA and with 250 s exposure time per frame; the detector-to-sample distance was 6 cm. Proxidecagonite is orthorhombic, space group *Pnma*, with unit-cell parameters: *a* = 29.013(3), *b* = 8.156(1), *c* = 12.401(2) Å, *V* = 2934.4(7) Å^3^ and *Z* = 4.

Single-crystal X-ray diffraction intensity data of proxidecagonite were integrated and corrected for standard Lorentz polarization factors with the *CrysAlis* RED package^30^. The program ABSPACK in *CrysAlis* RED^30^ was used for the absorption correction. Statistical tests (|*E*^2^ − 1| = 1.091) and systematic absences agrees with the space group *Pnma*. The crystal structure was refined, by means of the program SHELXL^31^, starting from the atomic coordinates reported for the orthorhombic approximant *o*’-Al_13_Co_4_^19^, which exhibits a similar unit-cell and the same space group. A least-squares refinement on *F*^2^ using these heavy-atom positions and isotropic temperature factors produced an *R* factor of ~10%. We did not observe any partially occupied/split positions in our model. The occupancy of all the sites was left free to vary (Ni vs. vacancy and Al vs. vacancy). All the structural sites were found to be consistent with a pure occupation by Ni and Al, respectively, and then fixed to the resulting value. Given the close scattering value between Ni and Fe, we refined all the Ni positions using the Ni curve. Neutral scattering curves for Ni and Al were taken from the *International Tables for X-ray Crystallography*^32^. At the last stage, with isotropic atomic displacement parameters for all atoms and no constraints, the residual value settled at *R*1 = 2.46% for 2360 observed reflections [*F*_o_ > 4σ(*F*_o_) level] and 110 parameters and at *R*1 = 3.03% for all 4537 independent reflections. Inspection of the difference Fourier map revealed that maximum positive and negative peaks were 0.63 and 1.33 e^−^/Å^3^, respectively. The formula obtained from the structure refinement (neglecting the Ni-Fe difference), Al_34_Ni_11_, is in excellent agreement with that obtained from electron microprobe data Al_33.99_Ni_9.00_Fe_1.98_Si_0.02_Co_0.01_.

Table S1 reports details of the selected crystal, data collection, and refinement. Atomic coordinates and equivalent isotropic displacement parameters are given in Table S2 whereas selected bond distances are given in Table S3.

Crystallographic data (CCDC 1851139) can be obtained free of charge from *The Cambridge Crystallographic Data Centre* via www.ccdc.cam.ac.uk/data_request/cif.

### X-ray powder diffraction

X-ray powder diffraction data (Table S4) were obtained with an Oxford Diffraction Xcalibur PX Ultra diffractometer fitted with a 165 mm diagonal Onyx CCD detector and using copper radiation (Cu*K*α, λ = 1.54138 Å). The working conditions were 40 kV × 45 nA with 4 hours of exposure; the detector-to-sample distance was 7 cm. The program *Crysalis* RED^30^ was used to convert the observed diffraction rings to a conventional powder diffraction pattern. The least squares refinement gave the following unit-cell values: *a* = 28.861(2), *b* = 8.1335(7), *c* = 12.3442(9) Å, and *V* = 2897.7(3) Å^3^.

### Electronic structure calculations

To assess the distribution of electronic charge within the system and the electronic features that affect the atomic structure, Bader charge^33^ and densities of states (DOS) were calculated using first-principles methods in the Vienna *ab initio* simulation package (VASP 5.4.4)^34–36^. The VASP setup utilizes plane waves with energy cutoffs at 500 eV and a self-consistent convergence limit criterion of 0.01 meV. The exchange-correlation potential is approximated non-locally by treating the electron densities as well as their gradients as the basis set in the generalized-gradient approximant (GGA) as constructed by Perdew, Burke, and Ernzerhof (PBE)^37^ and the orbital basis set includes Ni (3*d*^8^4*s*^2^) and Al (3*s*^2^3*p*^1^) for a total of 848 electrons in “Ni_44_Al_136_”. To rationalize which site might prefer Fe to Ni, Bader charge was also calculated for “Co_44_Al_136_”, for a total of 804 electrons (Table S5). In the Bader charge calculation of “Co_44_Al_136_”, sites that are more charge-rich prefer Ni to Fe. The DOS was plotted using 3001 *k*-points from an automatic mesh in the energy region −8– +2 eV.

## Electronic supplementary material

Supplementary Information
