# Supplementary material for: Previously unknown quasicrystal periodic approximant found in space
Source: Sci Rep. 2018 Nov 2;8:16271. doi: 10.1038/s41598-018-34375-x (PMC6214956; doi:10.1038/s41598-018-34375-x)
Supplement: Supplementary file 1 — Supplementary Information [file 41598_2018_34375_MOESM1_ESM.docx]

**Supplementary Information**

**Previously unknown quasicrystal periodic approximant found in space**

**Luca Bindi^1,^*, Joyce Pham^2^, Paul J. Steinhardt^3,4^**

^1^Dipartimento di Scienze della Terra, Università di Firenze, Via La Pira 4, I-50121 Florence, Italy

^2^Max-Planck-Institut für Chemische Physik fester Stoffe, Nöthnitzer Straße 40, 01187 Dresden, Germany

^3^Department of Physics, Princeton University, Jadwin Hall, Princeton, NJ-08544, USA

^4^Princeton Center for Theoretical Science, Princeton University, Princeton, NJ-08544, USA

*Corresponding Author: [luca.bindi@unifi.it](mailto:luca.bindi@unifi.it)

Table *S*1. Data and experimental details for the selected proxidecagonite crystal

|  |  |
| --- | --- |
|  |  |
| **Crystal data** | |
|  |  |
| Ideal formula | Al_34_Ni_9_Fe_2_ |
| Crystal size (mm^3^) | 0.022 × 0.019 × 0.016 |
| Form | block |
| Colour | black |
| Crystal system | orthorhombic |
| Space group | *Pnma* |
| *a* (Å) | 29.013(3) |
| *b* (Å) | 8.156(1) |
| *c* (Å) | 12.401(2) |
| *V* (Å^3^) | 2934.4(7) |
| *Z* | 4 |
|  |  |
| **Data collection** | |
|  |  |
| Instrument | Oxford Diffraction Xcalibur 3 |
| Radiation type | Mo*K*α (λ = 0.71073 Å) |
| Temperature (K) | 293(3) |
| Detector to sample distance (cm) | 6 |
| Number of frames | 2053 |
| Measuring time (s) | 250 |
| Maximum covered 2θ (°) | 59.95 |
| Absorption correction | multi-scan (Oxford Diffraction, 2006) |
| Collected reflections | 92563 |
| Unique reflections | 4537 |
| Reflections with *F*_o_ > 4σ(*F*_o_) | 2360 |
| *R*_int_ | 0.0445 |
| *R*_σ_ | 0.0512 |
| Range of *h*, *k*, *l* | -40 ≤ *h* ≤ 40, -11 ≤ *k* ≤ 11, -17 ≤ *l* ≤ 17 |
|  |  |
| *Refinement* | |
|  |  |
| Refinement | Full-matrix least squares on *F*^2^ |
| Final *R*_1_ [*F*_o_ > 4σ(*F*_o_)] | 0.0246 |
| Final *R*_1_ (all data) | 0.0303 |
| *S* | 0.987 |
| Number refined parameters | 110 |
| Δ*ρ*_max_ (e Å^-3^) | 0.63 |
| Δ*ρ*_min_ (e Å^-3^) | -1.33 |
|  |  |

Table *S*2. Atoms, fractional atomic coordinates and isotropic displacement parameters (Å^2^) for the selected proxidecagonite crystal.

| Atom | *x* | *y* | *z* | *U*_iso_ |
| --- | --- | --- | --- | --- |
| Ni1 | 0.214401(13) | 0.02807(5) | 0.43500(3) | 0.04320(10) |
| Ni2 | 0.167580(19) | ¼ | 0.74312(4) | 0.04845(14) |
| Ni3 | 0.079927(18) | ¼ | 0.44936(4) | 0.05008(15) |
| Ni4 | 0.419736(17) | ¼ | 0.92167(4) | 0.04193(13) |
| Ni5 | 0.167875(16) | ¼ | 0.13553(4) | 0.03713(11) |
| Ni6 | 0.318930(16) | ¼ | 0.26194(4) | 0.03405(11) |
| Ni7 | 0.030249(14) | 0.02789(5) | 0.74244(3) | 0.04959(10) |
| Ni8 | 0.420735(16) | ¼ | 0.56980(4) | 0.03884(12) |
| Ni9 | 0.077896(15) | ¼ | 0.04610(4) | 0.03573(12) |
| Al1 | 0.32157(3) | ¼ | 0.61251(8) | 0.0248(2) |
| Al2 | 0.37380(3) | ¼ | 0.09206(7) | 0.0271(2) |
| Al3 | 0.21565(2) | 0.03231(10) | 0.04978(6) | 0.03012(17) |
| Al4 | 0.03313(3) | 0.03263(10) | 0.13572(6) | 0.03438(18) |
| Al5 | 0.38012(3) | 0.01789(9) | 0.26183(6) | 0.03569(19) |
| Al6 | 0.38399(3) | ¼ | 0.75395(8) | 0.0240(2) |
| Al7 | 0.36652(3) | ¼ | 0.43119(7) | 0.01992(18) |
| Al8 | 0.31222(2) | 0.02975(9) | 0.74986(6) | 0.02842(15) |
| Al9 | 0.37361(3) | 0.01855(10) | 0.91981(7) | 0.0424(2) |
| Al10 | 0.43537(3) | 0.02846(10) | 0.43218(6) | 0.03611(18) |
| Al11 | 0.03301(2) | 0.03161(8) | 0.35586(5) | 0.01877(13) |
| Al12 | 0.10054(3) | ¼ | 0.24043(7) | 0.02057(19) |
| Al13 | 0.30268(2) | 0.02339(8) | 0.12242(5) | 0.02354(14) |
| Al14 | 0.43559(3) | 0.02901(10) | 0.05979(6) | 0.03358(18) |
| Al15 | 0.20803(3) | 0.02889(9) | 0.82089(6) | 0.03187(17) |
| Al16 | 0.15705(4) | ¼ | 0.94186(9) | 0.0394(3) |
| Al17 | 0.11920(3) | 0.00072(10) | 0.09051(7) | 0.0412(2) |
| Al18 | 0.45597(4) | ¼ | 0.24549(10) | 0.0479(3) |
| Al19 | 0.28074(3) | ¼ | 0.93238(8) | 0.0271(2) |
| Al20 | 0.23852(4) | ¼ | 0.66150(9) | 0.0353(3) |
| Al21 | 0.26713(4) | ¼ | 0.44039(9) | 0.0387(3) |
| Al22 | 0.47154(3) | ¼ | 0.74956(7) | 0.02063(19) |
| Al23 | 0.00604(3) | ¼ | 0.53222(10) | 0.0333(3) |
|  |  |  |  |  |

Table *S*3. Bond distances (in Å) in the structure of proxidecagonite

| Ni1—Al19 | 2.2726(5) | Al2—Ni4 | 2.4982(11) | Al12—Al17 (x2) | 2.8077(10) |
| --- | --- | --- | --- | --- | --- |
| Ni1—Al21 | 2.3708(8) | Al2—Al14 (x2) | 2.5735(11) | Al12—Al4 | 2.9418(11) |
| Ni1—Al13 | 2.4132(8) | Al2—Al13 (x2) | 2.7957(10) |  |  |
| Ni1—Al8 | 2.4678(8) | Al2—Al5 (x2) | 2.8372(10) | Al13—Ni1 | 2.4132(8) |
| Ni1—Al3 | 2.5273(8) | Al2—Al9 (x2) | 2.8507(11) | Al13—Al15 | 2.5172(10) |
| Ni1—Al9 | 2.5885(10) |  |  | Al13—Al20 | 2.5759(9) |
| Ni1—Al15 | 2.6986(9) | Al3—Ni1 | 2.5273(8) | Al13—Ni2 | 2.8209(8) |
|  |  | Al3—Al8 | 2.6583(10) |  |  |
| Ni2—Al20 | 2.2935(12) | Al3—Al1 | 2.6595(9) | Al14—Al9 | 2.5010(12) |
| Ni2—Al15 (x2) | 2.3579(9) | Al3—Al13 | 2.6817(10) | Al14—Ni7 | 2.5155(9) |
| Ni2—Al16 | 2.4834(12) | Al3—Al21 | 2.7187(10) | Al14—Ni4 | 2.5287(9) |
| Ni2—Al5 (x2) | 2.5967(9) | Al3—Al16 | 2.7989(12) | Al14—Ni3 | 2.6938(9) |
| Ni2—Al13 (x2) | 2.8209(8) | Al3—Al15 | 2.8471(11) | Al14—Al11 | 2.7332(9) |
|  |  | Al3—Al17 | 2.8552(12) | Al14—Al23 | 2.8571(10) |
| Ni3—Al23 | 2.3773(11) | Al3—Al19 | 2.9728(11) | Al14—Al23 | 2.9543(12) |
| Ni3—Al11 (x2) | 2.5239(8) | Al3—Al20 | 2.9982(10) | Al14—Al18 | 2.9835(13) |
| Ni3—Al9 (x2) | 2.5979 (9) |  |  |  |  |
| Ni3—Al12 | 2.6591(11) | Al4—Ni7 | 2.4305(9) | Al15—Ni6 | 2.5140(8) |
| Ni3—Al14 (x2) | 2.6938(9) | Al4—Al17 | 2.5726(13) | Al15—Al13 | 2.5172(10) |
|  |  | Al4—Al22 | 2.7064(10) | Al15—Al5 | 2.6875(12) |
| Ni4—Al9 (x2) | 2.3141(9) | Al4—Al11 | 2.7299(10) | Al15—Ni1 | 2.6986(9) |
| Ni4—Al6 | 2.3242(10) | Al4—Al10 | 2.7303(10) | Al15—Al16 | 2.7731(11) |
| Ni4—Al2 | 2.4982(11) | Al4—Ni8 | 2.7881(8) | Al15—Al21 | 2.8088(10) |
| Ni4—Al14 (x2) | 2.5287(9) | Al4—Al12 | 2.9418(11) | Al15—Al20 | 2.8181(11) |
| Ni4—Al23 | 2.5683(11) | Al4—Al10 | 2.9588(12) | Al15—Al3 | 2.8471(11) |
| Ni4—Al22 | 2.6103(10) |  |  |  |  |
| Ni4—Al11 (x2) | 2.7967(7) | Al5—Ni2 | 2.5967(9) | Al16—Ni5 | 2.4222(12) |
|  |  | Al5—Ni7 | 2.6381(10) | Al16—Ni9 | 2.6354(12) |
| Ni5—Al12 | 2.3471(10) | Al5—Al10 | 2.6533(11) | Al16—Al15 | 2.7731(12) |
| Ni5—Al16 | 2.4222(12) | Al5—Al15 | 2.6875(12) | Al16—Al3 (x2) | 2.7989(12) |
| Ni5—Al3 (x2) | 2.4909(8) | Al5—Al13 | 2.8354(11) | Al16—Al17 (x2) | 2.9559(11) |
| Ni5—Al17 (x2) | 2.5376(9) | Al5—Al7 | 2.8549(10) |  |  |
| Ni5—Al8 (x2) | 2.7476(8) | Al5—Al18 | 2.9099(12) | Al17—Ni8 | 2.3643(9) |
| Ni5—Ni9 | 2.8364(7) | Al5—Al14 | 2.9792(11) | Al17—Al10 | 2.5335(12) |
|  |  |  |  | Al17—Al1 | 2.6849(11) |
| Ni6—Al7 | 2.5122(10) | Al6—Al22 | 2.5406(14) | Al17—Al8 | 2.8152(12) |
| Ni6—Al15 (x2) | 2.5140(8) | Al6—Al8 (x2) | 2.7504(11) | Al17—Al7 | 2.8734(11) |
| Ni6—Al13 (x2) | 2.5752(8) | Al6—Al9 (x2) | 2.8080(11) | Al17—Al6 | 2.8806(11) |
| Ni6—Al5 (x2) | 2.5952(9) | Al6—Al17 (x2) | 2.8806(11) | Al17—Al16 | 2.9559(11) |
| Ni6—Al2 | 2.6405(11) |  |  |  |  |
| Ni6—Al21 | 2.6751(12) | Al7—Al10 (x2) | 2.6937(11) | Al18—Ni7 (x2) | 2.3018(5) |
|  |  | Al7—Al5 | 2.8549(10) | Al18—Al5 | 2.9099(12) |
| Ni7—Al11 | 2.2560(7) | Al7—Al17 (x2) | 2.8734(11) | Al18—Al14 | 2.9835(13) |
| Ni7—Al18 | 2.3018(5) | Al7—Al21 | 2.8858(14) | Al18—Al10 | 2.9970(13) |
| Ni7—Al4 | 2.4305(9) |  |  |  |  |
| Ni7—Al22 | 2.4886(7) | Al8—Ni1 | 2.4678(8) | Al19—Ni1 (x2) | 2.2726(5) |
| Ni7—Al14 | 2.5155(9) | Al8—Al3 | 2.6583(10) | Al19—Al3 (x2) | 2.9728(11) |
| Ni7—Al10 | 2.5966(9) | Al8—Ni5 | 2.7476(8) |  |  |
| Ni7—Al5 | 2.6381(10) | Al8—Al9 | 2.7608(12) | Al20—Al13 (x2) | 2.5759(9) |
|  |  | Al8—Al17 | 2.8152(12) | Al20—Al15 | 2.8181(11) |
| Ni8—Al7 | 2.3301(10) |  |  | Al20—Al21 | 2.8649(16) |
| Ni8—Al17 (x2) | 2.3643(9) | Al9—Al14 | 2.5010(12) | Al20—Al3 (x2) | 2.9982(10) |
| Ni8—Al6 | 2.5202(11) | Al9—Ni1 | 2.5885(10) |  |  |
| Ni8—Al10 (x2) | 2.5215(9) | Al9—Ni3 | 2.5979(9) | Al21—Ni1 | 2.3708(8) |
| Ni8—Al22 | 2.6724(10) | Al9—Al2 | 2.8507(11) | Al21—Al3 (x2) | 2.7187(10) |
| Ni8—Al4 (x2) | 2.7881(8) | Al9—Al11 | 2.8526(11) | Al21—Al15 (x2) | 2.8088(10) |
| Ni8—Al1 | 2.9254(11) |  |  |  |  |
|  |  | Al10—Al17 | 2.5335(12) | Al22—Ni7 (x2) | 2.4886(7) |
| Ni9—Al17 (x2) | 2.4234(9) | Al10—Ni7 | 2.5966(9) | Al22—Al11 (x2) | 2.6515(8) |
| Ni9—Al4 (x2) | 2.4628(9) | Al10—Ni9 | 2.7022(9) | Al22—Al4 (x2) | 2.7064(10) |
| Ni9—Al12 | 2.4978(10) | Al10—Al4 | 2.7303(10) | Al22—Al23ix | 2.8853(15) |
| Ni9—Al16 | 2.6354(12) | Al10—Al4 | 2.9588(12) |  |  |
| Ni9—Al10 (x2) | 2.7022(9) | Al10—Al18 | 2.9970(13) | Al23—Ni4 | 2.5684(11) |
|  |  |  |  | Al23—Al14 (x2) | 2.8571(10) |
| Al1—Al20 | 2.4851(15) | Al11—Ni7 | 2.2560(7) | Al23—Al22 | 2.8853(15) |
| Al1—Al8 (x2) | 2.4903(10) | Al11—Al22 | 2.6515(8) | Al23—Al11 (x2) | 2.9129(10) |
| Al1—Al6 | 2.5211(13) | Al11—Al14 | 2.7332(9) | Al23—Al11 | 2.9271(12) |
| Al1—Al7 | 2.5993(13) | Al11—Ni4 | 2.7967(7) | Al23—Al14 (x2) | 2.9543(12) |
| Al1—Al21 | 2.6554(15) | Al11—Al9 | 2.8526(11) |  |  |
| Al1—Al3 (x2) | 2.6595(9) | Al11—Al23 | 2.9129(10) |  |  |
| Al1—Al17 (x2) | 2.6849(11) | Al11—Al23 | 2.9271(12) |  |  |

Table *S*4. Measured and calculated X-ray powder diffraction data (*d* in Å) for proxidecagonite. The strongest diffraction lines are given in bold.

| *hkl* | *d*_meas_ | *I*_meas_ | *d*_calc_ | *I*_calc_ |
| --- | --- | --- | --- | --- |
| 301 | - | - | 7.5932 | 4 |
| 401 | **-** | **-** | 6.2341 | 3 |
| 411 | - | - | 4.9489 | 4 |
| 511 | - | - | 4.4017 | 4 |
| 502 | - | - | 4.2184 | 4 |
| 203 | **3.96** | **50** | 3.9578 | 51 |
| 602 | **3.80** | **40** | 3.7966 | 45 |
| 800 | 3.609 | 15 | 3.6112 | 14 |
| 321 | 3.588 | 10 | 3.5865 | 14 |
| 420 | 3.541 | 20 | 3.5451 | 24 |
| 801 | - | - | 3.4660 | 11 |
| 421 | **3.403** | **40** | 3.4074 | 35 |
| 122 | **-** | **-** | 3.3741 | 5 |
| 222 | 3.310 | 20 | 3.3071 | 23 |
| 521 | - | - | 3.2123 | 3 |
| 603 | - | - | 3.1284 | 9 |
| 901 | - | - | 3.1067 | 3 |
| 622 | - | - | 2.7759 | 4 |
| 623 | **-** | **-** | 2.4801 | 5 |
| 630 | - | - | 2.3634 | 6 |
| 432 | - | - | 2.3485 | 8 |
| 532 | - | - | 2.2816 | 4 |
| 032 | - | - | 2.2649 | 4 |
| 433 | - | - | 2.1611 | 6 |
| 615 | **-** | **-** | 2.1212 | 6 |
| 1302 | - | - | 2.0909 | 4 |
| 1220 | **2.069** | **50** | 2.0720 | 45 |
| 006 | 2.058 | 20 | 2.0577 | 19 |
| 106 | - | - | 2.0525 | 4 |
| 832 | - | - | 2.0463 | 4 |
| 1023 | **2.045** | **100** | 2.0444 | 100 |
| 805 | 2.036 | 30 | 2.0383 | 33 |
| 040 | **2.033** | **50** | 2.0345 | 49 |
| 425 | **2.024** | **70** | 2.0262 | 77 |
| 1410 | - | - | 2.0003 | 3 |
| 1030 | - | - | 1.9775 | 7 |
| 1402 | 1.955 | 20 | 1.9571 | 24 |
| 833 | - | - | 1.9187 | 7 |
| 243 | - | - | 1.8094 | 9 |
| 642 | - | - | 1.7933 | 8 |
| 608 | - | - | 1.4696 | 6 |
| 1226 | - | - | 1.4600 | 4 |
| 1605 | - | - | 1.4575 | 5 |
| 1822 | - | - | 1.4512 | 5 |
| 046 | - | - | 1.4467 | 9 |
| 2000 | - | - | 1.4445 | 3 |
| 845 | - | - | 1.4399 | 13 |
| 1442 | - | - | 1.4105 | 11 |
| 2203 | - | - | 1.2510 | 10 |
| 1408 | - | - | 1.2359 | 8 |
| 0010 | - | - | 1.2346 | 3 |
| 648 | - | - | 1.1913 | 4 |
| 1645 | - | - | 1.1848 | 4 |
| 1260 | - | - | 1.1817 | 3 |
| 1063 | - | - | 1.1765 | 8 |
| 465 | - | - | 1.1730 | 5 |
| 1828 | - | - | 1.0731 | 5 |
| 4211 | - | - | 1.0700 | 4 |
| 2243 | - | - | 1.0657 | 8 |
| 1448 | - | - | 1.0563 | 7 |
| 2622 | - | - | 1.0561 | 3 |
| 2425 | - | - | 1.0457 | 3 |

*Note*: calculated diffraction pattern obtained with the atom coordinates reported in Table 4 (only reflections with *I*_rel_ ≥ 3 are listed).

**Bader effective charges.** In Bader charge analysis, the total number of electrons (848 *e^–^* in Ni_44_Al_136_ or 804 *e^–^* in “Co_44_Al_136_”) of a system is distributed to the atomic sites based on their chemical potentials, which includes their relative electronegativity differences, or the ability of an atom to attract electrons to itself. In general, from Bader charge analysis, Ni sites are all negatively charged with an average of –2.75 and Al sites are mostly positively charged with an average of +0.89. This charge separation may indicate that “Ni_44_Al_136_” is a “nickelide”, where Ni atoms uptake electrons from the relatively more electropositive Al atoms. Both Ni and Al sites with the greatest negative charges reside at the vertex-sharing corners along the *b*-axis of the trigonal bipyramids.

Table *S*5. Bader charges in Ni_44_Al_136_ and in “Co_44_Al_136_”

| Refinement | | Bader Charge | | “Oxidation” |
| --- | --- | --- | --- | --- |
|  |  | “Co_44_Al_136_” | Ni_44_Al_136_ | Charge |
| Ni1 | 8*d* | 11.85 | 12.63 | –2.63 |
| Ni2 | 4*c* | 11.94 | 12.63 | –2.63 |
| Ni3 | 4*c* | 11.48 | 12.27 | –2.27 |
| Ni4 | 4*c* | 13.32 | 13.96 | –3.96 |
| Ni5 | 4*c* | 11.38 | 12.30 | –2.30 |
| Ni6 | 4*c* | 11.49 | 12.36 | –2.36 |
| Ni7 | 8*d* | 12.06 | 12.92 | –2.92 |
| Ni8 | 4*c* | 12.41 | 13.16 | –3.16 |
| Ni9 | 4*c* | 11.55 | 12.50 | –2.50 |
| Al1 | 4*c* | 4.951 | 4.627 | –1.627 |
| Al2 | 4*c* | 2.506 | 2.624 | +0.376 |
| Al3 | 8*d* | 2.116 | 2.146 | +0.854 |
| Al4 | 8*d* | 1.839 | 1.899 | +1.101 |
| Al5 | 8*d* | 2.280 | 2.281 | +0.719 |
| Al6 | 4*c* | 1.803 | 1.891 | +1.109 |
| Al7 | 4*c* | 1.892 | 1.918 | +1.082 |
| Al8 | 8*d* | 2.277 | 2.281 | +0.719 |
| Al9 | 8*d* | 1.805 | 1.860 | +1.140 |
| Al10 | 8*d* | 2.188 | 2.237 | +0.763 |
| Al11 | 8*d* | 1.700 | 1.797 | +1.203 |
| Al12 | 4*c* | 1.869 | 1.931 | +1.069 |
| Al13 | 8*d* | 2.093 | 2.168 | +0.832 |
| Al14 | 8*d* | 2.024 | 2.108 | +0.892 |
| Al15 | 8*d* | 1.874 | 1.999 | +1.001 |
| Al16 | 4*c* | 1.821 | 1.902 | +1.098 |
| Al17 | 8*d* | 1.730 | 1.758 | +1.242 |
| Al18 | 4*c* | 1.632 | 1.743 | +1.257 |
| Al19 | 4*c* | 1.560 | 1.718 | +1.282 |
| Al20 | 4*c* | 2.152 | 2.516 | +0.484 |
| Al21 | 4*c* | 1.880 | 1.919 | +1.081 |
| Al22 | 4*c* | 1.719 | 1.806 | +1.194 |
| Al23 | 4*c* | 1.980 | 2.061 | +0.939 |
